# Association of age with perioperative morbidity among patients undergoing surgical management of minor burns

**DOI:** 10.3389/fsurg.2023.1131293

**Published:** 2023-02-27

**Authors:** Samuel Knoedler, Dany Y. Matar, Leonard Knoedler, Doha Obed, Valentin Haug, Sabina M. Gorski, Bong-Sung Kim, Martin Kauke-Navarro, Ulrich Kneser, Adriana C. Panayi, Dennis P. Orgill, Gabriel Hundeshagen

**Affiliations:** ^1^ Department of Plastic, Hand and Reconstructive Surgery, University Hospital Regensburg, Regensburg, Germany; ^2^ Division of Plastic Surgery, Department of Surgery, Brigham and Women’s Hospital, Harvard Medical School, Boston, MA, United States; ^3^ Division of Plastic and Reconstructive Surgery, Massachusetts General Hospital, Harvard Medical School, Boston, MA, United States; ^4^ Department of Plastic, Aesthetic, Hand and Reconstructive Surgery, Hannover Medical School, Hannover, Germany; ^5^ Department of Hand-, Plastic and Reconstructive Surgery, Burn Center, BG Trauma Center Ludwigshafen, University of Heidelberg, Ludwigshafen, Germany; ^6^ Department of Plastic Surgery and Hand Surgery, University Hospital Zurich, Zurich, Switzerland; ^7^ Division of Plastic Surgery, Department of Surgery, Yale New Haven Hospital, Yale School of Medicine, New Haven, CT, United States

**Keywords:** burn surgery, burns, burn injury, minor burns, age, elderly, geriatric

## Abstract

**Introduction:**

Burn injuries are associated with significant morbidity, often necessitating surgical management. Older patients are more prone to burns and more vulnerable to complications following major burns. While the relationship between senescence and major burns has already been thoroughly investigated, the role of age in minor burns remains unclear. To better understand differences between elderly and younger patients with predominantly minor burns, we analyzed a multi-institutional database.

**Methods:**

We reviewed the 2008-2020 ACS-NSQIP database to identify patients who had suffered burns according to ICD coding and underwent initial burn surgery.

**Results:**

We found 460 patients, of which 283 (62%) were male and 177 (38%) were female. The mean age of the study cohort was 46 ± 17 years, with nearly one-fourth (*n* = 108; 23%) of all patients being aged ≥60 years. While the majority (*n* = 293; 64%) suffered from third-degree burns, 22% (*n* = 99) and 15% (*n* = 68) were diagnosed with second-degree burns and unspecified burns, respectively. An average operation time of 46 min, a low mortality rate of 0.2% (*n* = 1), a short mean length of hospital stay (1 day), and an equal distribution of in- and outpatient care (51%, *n* = 234 and 49%, *n* = 226, respectively) indicated that the vast majority of patients suffered from minor burns. Patients aged ≥60 years showed a significantly prolonged length of hospital stay (*p*<0.0001) and were significantly more prone to non-home discharge (*p*<0.0001). In univariate analysis, advanced age was found to be a predictor of surgical complications (*p* = 0.001) and medical complications (*p* = 0.0007). Elevated levels of blood urea nitrogen (*p*>0.0001), creatinine (*p*>0.0001), white blood cell count (*p*=0.02), partial thromboplastin time (*p* = 0.004), and lower levels of albumin (*p* = 0.0009) and hematocrit (*p*>0.0001) were identified as risk factors for the occurrence of any complication. Further, complications were more frequent among patients with lower body burns.

**Discussion:**

In conclusion, patients ≥60 years undergoing surgery for predominantly minor burns experienced significantly more complications. Minor lower body burns correlated with worse outcomes and a higher incidence of adverse events. Decreased levels of serum albumin and hematocrit and elevated values of blood urea nitrogen, creatinine, white blood count, and partial thromboplastin time were identified as predictive risk factors for complications.

## 1. Introduction

Burns injuries can be devastating and are associated with high morbidity and mortality. In the United States, more than 1 million burn injuries and up to 3,500 burn-related deaths are recorded annually (1, 2). Burns are typically classified according to the percentage of total body surface area involvement (%TBSA) and the burn depth (3). In the traditional classification, first-degree burns are limited to the epidermis, second-degree burns extend to the dermal layer (superficial or partial thickness) and third-degree burns are marked by full-thickness skin burns and subcutaneous fat layer involvement. While first-degree and superficial second-degree burns are commonly treated non-surgically, deep second and third-degree burns often require surgical excision and skin grafting, e.g., autologous split-thickness skin grafts or synthetic skin replacement, in the immediate phase after the injury (4, 5).

It has been thoroughly demonstrated that the area and degree of burn directly correlate with patient morbidity and mortality (6–10). In this context, advanced age has been identified as a crucial risk factor, with geriatric burn patients showing worse survival rates and postoperative sequelae (11–15). It is, therefore, not surprising that common burn prognosis indexes, such as the abbreviated burn severity index and the Baux-score integrate patient's age as a determining factor for survival prediction (16, 17). Jeschke et al. found, that in elderly patients even relatively small burn injuries become increasingly life-threatening and, therefore, require adequate (surgical) care (18). Accordingly, in a 2020 study, Goei et al. reported significantly higher burn surgery rates among elderly burn patients while reiterating their particular vulnerability (15).

To date, however, knowledge of burn surgery outcomes has been widely derived from retrospective analyses of single-institution experience, intervention-specific medical records, and/or cases of severe burn injuries. As a result, research significance and transferability are limited, with scarce evidence on burn surgery in elderly patients suffering from less drastic burn lesions. In 2020, the Committee on Elderly Burn Care acknowledged this paucity and called for an improvement in the identification and prediction of outcomes among elderly burn patients (11). By pooling patient data with geographical and institutional variance, the analysis of a multicenter database can help to fill this literature gap and provide an age-stratified overview of burn surgery outcomes also in patients with less-extensive injuries.

The American College of Surgeons National Surgical Quality Improvement Program (ACS-NSQIP) includes a diverse surgical patient population, capturing validated data from more than 700 hospitals. As a risk-adjusted and multi-institutional clinical registry, the ACS-NSQIP provides information on over 150 pre-, peri-, and post-operative variables for patients undergoing surgery. Selective audits and peer reviews ensure the quality, reliability, and accuracy of the information entered. Therefore, we queried this multi-institutional database to investigate perioperative outcomes and predictive risk factors of burn surgery, with a special focus on elderly patients.

## 2. Methods

### 2.1. Data source and patient selection

Data were collected between 2008 and 2020 from the ACS-NSQIP database. Originally, these data stem from 607 hospitals within the US and 100 hospitals in 11 other countries. Hospitals have a randomized process for patient selection and most of these hospitals are not designated burn centers. Currently, 35 of the 123 American burn centers (Supplementary Table S1) report patient data to the ACS-NSQIP. The records analyzed contain strictly de-identified information. Ethical approval to complete this retrospective analysis was obtained from the Brigham and Women's Hospital (Protocol #: 2013P001244).

**Figure 1:**
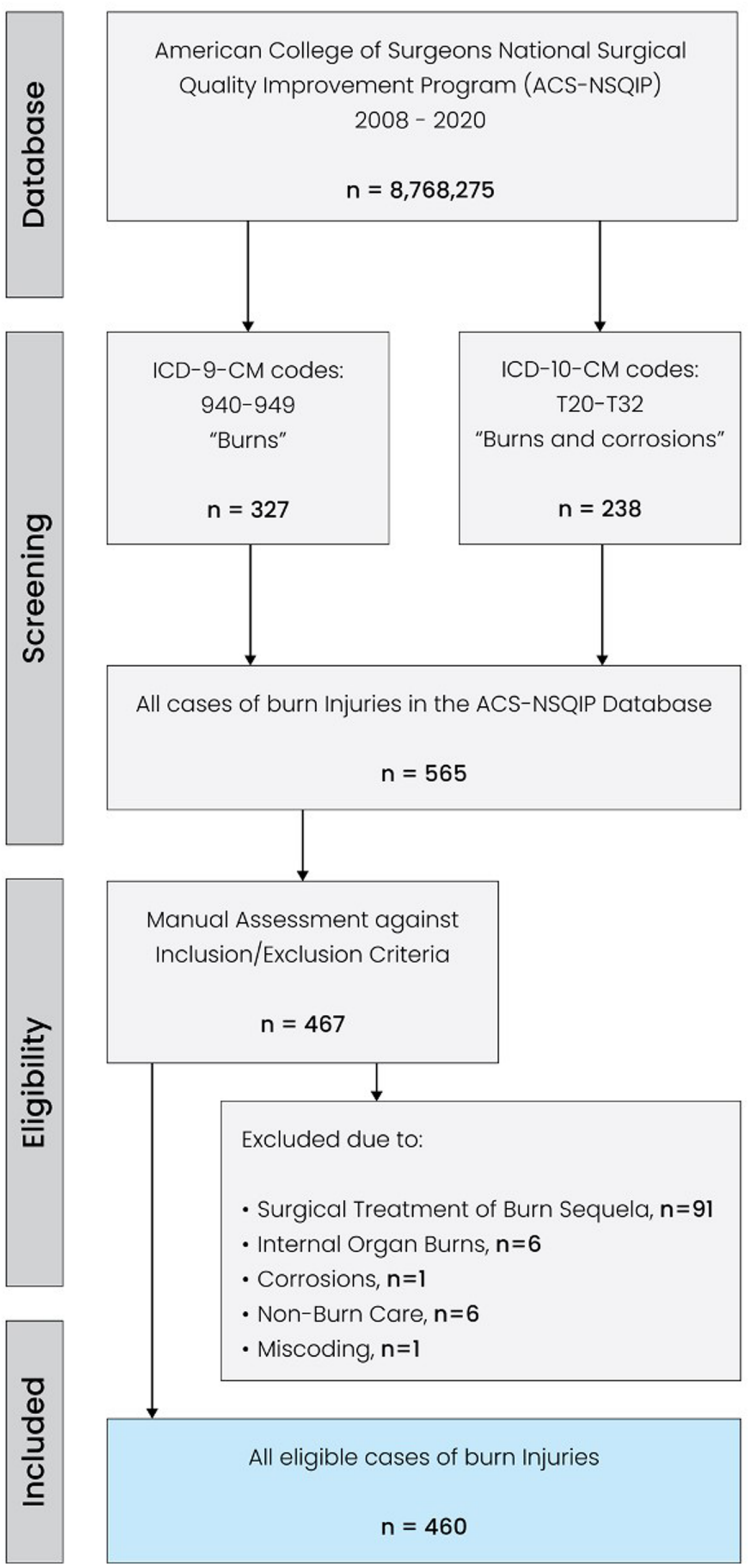
Flow diagram of the search and selection process.

The ACS-NSQIP database was queried to identify all burn patients who were diagnosed with burn injuries (*via* ICD coding) and underwent initial surgical management. The ACS-NSQIP is a database capturing surgical cases of patients aged 18 years and older. Therefore, *a priori*, non-surgical cases and pediatric or adolescent patients were not included in this study. 13 annual datasets between 2008 and 2020 were screened for the ICD-9-CM codes 940–949 (“Burns”) and ICD-10-CM codes T20-T32 (“Burns and corrosions”). Patients with other and/or more extensive diagnoses, such as polytrauma with concurrent burn injuries were excluded. This initial ICD-based search yielded 565 patient cases, which were then manually reviewed against the inclusion criteria by two investigators (SK and DYM). A third investigator (LK) was consulted to resolve any discrepant assessments. A total of 105 cases were excluded due to the following reasons: surgical treatment of burn sequela, internal organ burns, non-burn care, and corrosions. Thus, all cases with treatments beyond the scope of initial skin burn surgery and/or concurrent non-burn surgery interventions were excluded. In addition, one patient case with a body mass index below 7 kg/m^2^ was excluded due to miscoding. As a result, we compiled a homogeneous cohort of patients who had suffered isolated skin burns and underwent initial surgical management thereof. The flowchart illustrating the screening process is shown in Figure 1. The NSQIP utilizes the traditional description of burns (first, second, and third degree). To investigate the relevance and impact of age on morbidity and mortality in surgical burn care, we dichotomized the patient pool into patients aged ≥60 years and patients <60 years. In this regard, we followed previous burn-related studies and adopted the United Nations' threshold defining older people as those aged ≥60 years (19–21).

### 2.2. Variable extraction

We extracted pre-, peri-, and postoperative variables for analysis. In terms of pre- and perioperative data, we evaluated all variables that have been consistently recorded by the ACS-NSQIP throughout the 13-year study period: (i) patient demographics such as sex, age, race, height (inches), weight (pounds), body mass index, (ii) comorbidities (history of chronic obstructive pulmonary disease [COPD], congestive heart failure [CHF], diabetes mellitus, and disseminated cancer, hypertension requiring treatment, active dialysis therapy, dyspnea, nicotine abuse within 12 months prior to surgery, current use of corticosteroids, ventilator dependency, weight loss exceeding 10% of body weight, preoperative wound infections, and functional health status), (iii) preoperative scores (American Society of Anesthesiology (ASA) physical status classification [score 1–4]) and wound classification [score 1–4], and (iv) preoperative laboratory parameters including serum albumin, serum creatinine, serum sodium, serum glutamic-oxaloacetic transaminase (SGOT), alkaline phosphatase (AP), blood urea nitrogen (BUN), total bilirubin, white blood count (WBC), platelet count, hematocrit, international normalized ratio (INR), partial thromboplastin time (PTT), and prothrombin time (PT). Additionally, we evaluated the operative setting (in- or outpatient). Table 1 provides a detailed list of all retrieved pre- and perioperative variables.

**Table 1:** Patient demographics and comorbidities. Reported as *n* (%).

Characteristic	Total (*n* = 460)	<60 years (*n* = 352)	≥60 years (*n* = 108)
Demographics			
Sex			
Female (*n*)	177 (39)	137 (39)	40 (37)
Male (*n*)	283 (62)	215 (61)	68 (63)
Age (years), mean ± SD	46 ± 17	39 ± 12	70 ± 8.0
BMI (kg/m^2^), mean ± SD	29 ± 7.5	29 ± 7.8	29 ± 6.6
Race			
American Indian or Alaskan native	8 (1.7)	7 (2.0)	1 (0.9)
Asian	9 (2.0)	7 (2.0)	2 (1.9)
Native Hawaiian or Pacific Islander	3 (0.7)	2 (0.6)	1 (0.9)
Black or African American	76 (17)	60 (17)	16 (15)
White	329 (72)	249 (71)	80 (74)
Other or unknown	35 (7.6)	27 (7.6)	8 (7.4)
Preoperative Comorbidities			
Diabetes	88 (19)	58 (17)	30 (27)
Insulin treated diabetes	47 (10)	33 (9.4)	14 (13)
COPD	10 (2.2)	8 (2.2)	2 (1.9)
Obesity	170 (37)	129 (37)	41 (38)
CHF	4 (0.9)	3 (0.9)	1 (0.9)
Hypertension	146 (32)	97 (28)	49 (45)
Dyspnea	13 (2.8)	9 (2.6)	4 (3.7)
Current smoker	152 (33)	132 (38)	20 (19)
Corticosteroid use	11 (2.4)	8 (2.2)	3 (2.8)
Weight loss >10%	3 (0.7)	2 (0.6)	1 (0.9)
Disseminated cancer	1 (0.2)	1 (0.3)	0 (0.0)
Wound infection	202 (44)	155 (44)	47 (44)
ASA class			
1 – No disturbance	47 (10)	39 (11)	8 (7.4)
2 – Mild disturbance	257 (56)	202 (57)	55 (51)
3 – Severe disturbance	130 (28)	95 (27)	35 (32)
4 – Life-threatening	22 (4.8)	14 (4.0)	8 (7.4)
5 – Moribund	1 (0.2)	1 (0.3)	0 (0.0)
Wound class			
1 – Clean	94 (20)	68 (19)	26 (24)
2 – Clean/Contaminated	112 (24)	83 (24)	29 (27)
3 – Contaminated	129 (28)	100 (28)	29 (27)
4 – Dirty/Infected	125 (27)	96 (27)	29 (27)
Preoperative Laboratory Values			
Serum sodium (mmol/L), mean ± SD	138 ± 3.2	138 ± 3.0	138 ± 3.7
BUN (mg/dl), mean ± SD	15.5 ± 9.6	14 ± 9.4	19 ± 9.4
Creatinine (g/D), mean ± SD	1.1 ± 1.1	1.1 ± 1.1	1.2 ± 1.1
Serum albumin (g/dl), mean ± SD	3.5 ± 0.7	3.7 ± 0.7	3.3 ± 0.8
Total bilirubin (mg/dl), mean ± SD	0.6 ± 0.5	0.6 ± 0.5	0.6 ± 0.4
SGOT (U/L), mean ± SD	35 ± 40	35 ± 45	37 ± 42
Alkaline phosphatase (U/L), mean ± SD	92 ± 58	97 ± 65	80 ± 34
WBC (×10^3^/mm^3^), mean ± SD	9.2 ± 3.5	9.5 ± 3.5	8.5 ± 3.5
Hematocrit (% of RBCs), mean ± SD	38 ± 5.9	39 ± 5.7	37 ± 6.2
Platelet count (×10^3^/µl), mean ± SD	273 ± 110	281 ± 111	251 ± 103
PTT (sec), mean ± SD	33 ± 8.7	32 ± 9.2	34 ± 7.6
INR of PT values, mean ± SD	1.1 ± 0.5	1.1 ± 0.5	1.2 ± 0.2
PT (sec), mean ± SD	14 ± 3.0	13 ± 2.1	15 ± 3.7
Functional Status			
Independent	429 (93)	335 (95)	95 (81)
Partially or Totally Dependent	23 (5.0)	10 (2.8)	13 (12)
Setting			
Inpatient	234 (51)	159 (45)	75 (70)
Outpatient	226 (49)	193 (55)	33 (31)

As 30-day postoperative outcomes, we analyzed the discharge destination and the length of hospital stay (LOS). LOS is calculated as the difference in days between the date of admission and the date of discharge. Any complication was delineated as the occurrence of one of the following: Mortality, reoperation, readmission or unplanned readmission, surgical or medical complications. All surgical complications captured in the ACS-NSQIP database (i.e., superficial and deep incision site infections, organ space infections, wound dehiscences, and blood transfusions) were analyzed. Similarly, while considering all medical complications recorded in the ACS-NSQIP database, we concentrated on those that had occurred at least once. Details on postoperative outcomes and complications following burn surgery are shown in Tables 2, 3.

**Table 2:** Types of burn injury and type-specific occurrence of any complication. Reported as *n* (%).

Type of Burn	Total (*n* = 460)	Any Complication	Any Complication/Total (%)
Second Degree Burn	99 (22)		
Head and Neck	7 (1.5)	0	0.0
Upper Body	49 (11)	1	2.0
Lower Body	43 (9.3)	4	9.3
Unspecified Area	0 (0.0)	0	0.0
Third Degree Burn	293 (64)		
Head and Neck	13 (2.8)	2	15
Upper Body	143 (31)	21	15
Lower Body	124 (27)	29	23
Unspecified Area	13 (2.8)	1	7.7
Unspecified Degree Burn	68 (15)		
Head and Neck	8 (1.7)	0	0.0
Upper Body	19 (4.1)	5	26
Lower Body	15 (3.3)	4	27
Unspecified Area	26 (5.7)	7	27

**Table 3:** Comparison of outcomes following burn surgery between patients younger and older than 60 years of age.

Outcome	Total (*n* = 460)	<60 years (*n* = 352)	≥60 years (*n* = 108)	*P* value
Length of Hospital Stay, Median (IQR)	1 (0–8)	1 (0–5)	6 (0–13)	**<0.0001**
Operative time, Median (IQR)	46 (25–73)	46 (30–73)	40 (20–72)	0.12
Any Complication	74 (16)	49 (14)	25 (23)	**0.03**
Mortality within 30 days	1 (0.2)	0 (0.0)	1 (0.9)	0.23
Reoperation	22 (4.8)	15 (4.3)	7 (6.5)	0.44
Readmission	26 (5.7)	19 (5.4)	7 (6.5)	0.64
Unplanned Readmission	12 (2.6)	8 (2.3)	4 (3.7)	0.49
Surgical Complication	31 (6.7)	22 (6.3)	9 (8.3)	0.51
Superficial Incisional Infection	10 (2.2)	10 (2.8)	0 (0.0)	0.13
Deep Incisional Infection	4 (0.9)	4 (1.1)	0 (0.0)	0.58
Organ Space Infection	1 (0.2)	1 (0.3)	0 (0.0)	>0.99
Dehiscence	3 (0.7)	1 (0.3)	2 (1.9)	0.14
Blood Transfusions	13 (2.8)	6 (1.7)	7 (6.5)	**0.02**
Medical Complication	12 (2.6)	6 (1.7)	6 (5.6)	**0.04**
Pneumonia	4 (0.9)	2 (0.6)	2 (1.9)	0.24
Ventilator >48 h	4 (0.9)	1 (0.3)	3 (2.8)	**0.04**
Renal Insufficiency	1 (0.2)	1 (0.3)	0 (0.0)	>0.99
Urinary Tract Infection	1 (0.2)	1 (0.3)	0 (0.0)	>0.99
Deep Vein Thrombosis	1 (0.2)	1 (0.3)	0 (0.0)	>0.99
Sepsis	4 (0.9)	4 (1.1)	2 (1.9)	0.63
Septic Shock	2 (0.4)	2 (0.6)	1 (0.9)	0.55
Discharge destination				
Home	384 (84)	305 (87)	79 (73)	**0.002**
Not-Home	36 (7.8)	12 (3.4)	22 (20)	**<0.0001**
Other/unknown	40 (8.7)	33 (9.4)	7 (6.5)	0.44

Reported as *n* (%), unless otherwise stated. Statistically significant *p*-values are highlighted in bold.

### 2.3. Burn-specific parameters and variable extraction

We attempted to extract burn-specific variables. Since the %TBSA was not specified in the ACS-NSQIP database, the main classification was based on the burn degree. We refined this classification pattern by specifying which body area was affected by the burn injury (head and neck, upper body, lower body, unspecified area). When sorting and classifying each case, we closely adhered to the nomenclature and diagnostic details provided in the NSQIP database. Owing to the limited information recorded in the database, in some cases, a more accurate specification of the burn severity and the affected body part(s) was not possible. The classification scheme and the prevalence of each burn (sub)type are shown in Table 2.

### 2.4. Statistical analysis

The raw data of the ACS-NSQIP annual datasets were converted into analyzable Microsoft Excel (Version 16, Microsoft Corporation, Redmond, WA, USA) files *via* IBM SPSS Statistics for Windows, version 29 (IBM Corporation, Armonk, NY, USA). Subsequently, all ACS-NSQIP datasets between 2008 and 2020 were standardized into a consistent format. These data were collected and saved in an electronic laboratory notebook (LabArchives, LLC, San Marcos, CA, USA), and analyzed using GraphPad Prism (V9.00 for MacOS, GraphPad Software, La Jolla California, United States). We used independent *t*-tests to analyze continuous variables. The results were reported as means with standard deviations. Differences in categorical variables were calculated with Pearson's *χ*^2^ test. In cases with less than 10 events, Fisher's exact test was applied. To identify differences between groups with non-normally distributed data, we employed the Mann–Whitney *U*-test. The threshold for statistical significance was set at *p* < 0.05. After partitioning the cohort into three subgroups according to the occurrence of any, surgical, and medical complications, a univariate subgroup analysis was performed to identify risk factors for complications. All variables identified as significant predictors of the occurrence of any complication were included in a multivariate regression to compensate for confounding. For these confounder-adjusted results, we reported the odds ratio with 95% confidence interval to quantify the correlation between risk factors and outcomes.

## 3. Results

### 3.1. Patient demographics

The study population included 460 burn patients who underwent initial surgery over a 13-year review period between 2008 and 2020. The mean patient age and BMI were 46 ± 17 years and 29 ± 7.5 kg/m^2^, respectively. About one-fourth (*n* = 108; 23%) of all patients were aged ≥60. While male (*n* = 283; 62%) and white patients (*n* = 329; 72%) represented the majority of our patient cohort, preoperative wound infections (*n* = 202; 44%) and obesity (BMI > 30; *n* = 170; 37%) accounted for the most common comorbidities. Table 1 presents the demographic data and comorbidities of the total study population, all patients aged ≥60 years and all patients <60 years of age.

### 3.2. Surgical characteristics

The majority of patients suffered third-degree burns (*n* = 293; 64%), with the upper body affected in 143 (49%) and the lower body in 124 (42%) of cases (Table 2). In our patient cohort, second-degree burns accounted for 22% (*n* = 99) of cases. While our patient pool included no case of first-degree burn, the degree of burn remained unspecified in 68 (15%) cases. A nearly equal number of cases was performed in the outpatient setting (*n* = 226; 49%) and in the inpatient (*n* = 234; 51%) setting.

### 3.3. Perioperative outcomes

The median operative time was 46 (IQR: 25–73) minutes. Following a LOS of one day (IQR: 0–8), 384 (84%) patients were discharged home. The LOS was significantly different (*p* < 0.0001) between patients aged <60 years (1 day, IQR: 0–5) and those ≥60 years (6 days, IQR: 0–13). Significant differences were also found between the two age groups with regard to the discharge destination: While 305 (87%) patients aged <60 years and 79 (73%) patients ≥60 years were discharged home, 12 (3.4%) non-seniors and 22 (20%) seniors did not return to a home-based facility. Table 3 lists the perioperative outcomes in detail.

### 3.4. Postoperative surgical and medical outcomes

**Figure 2:**
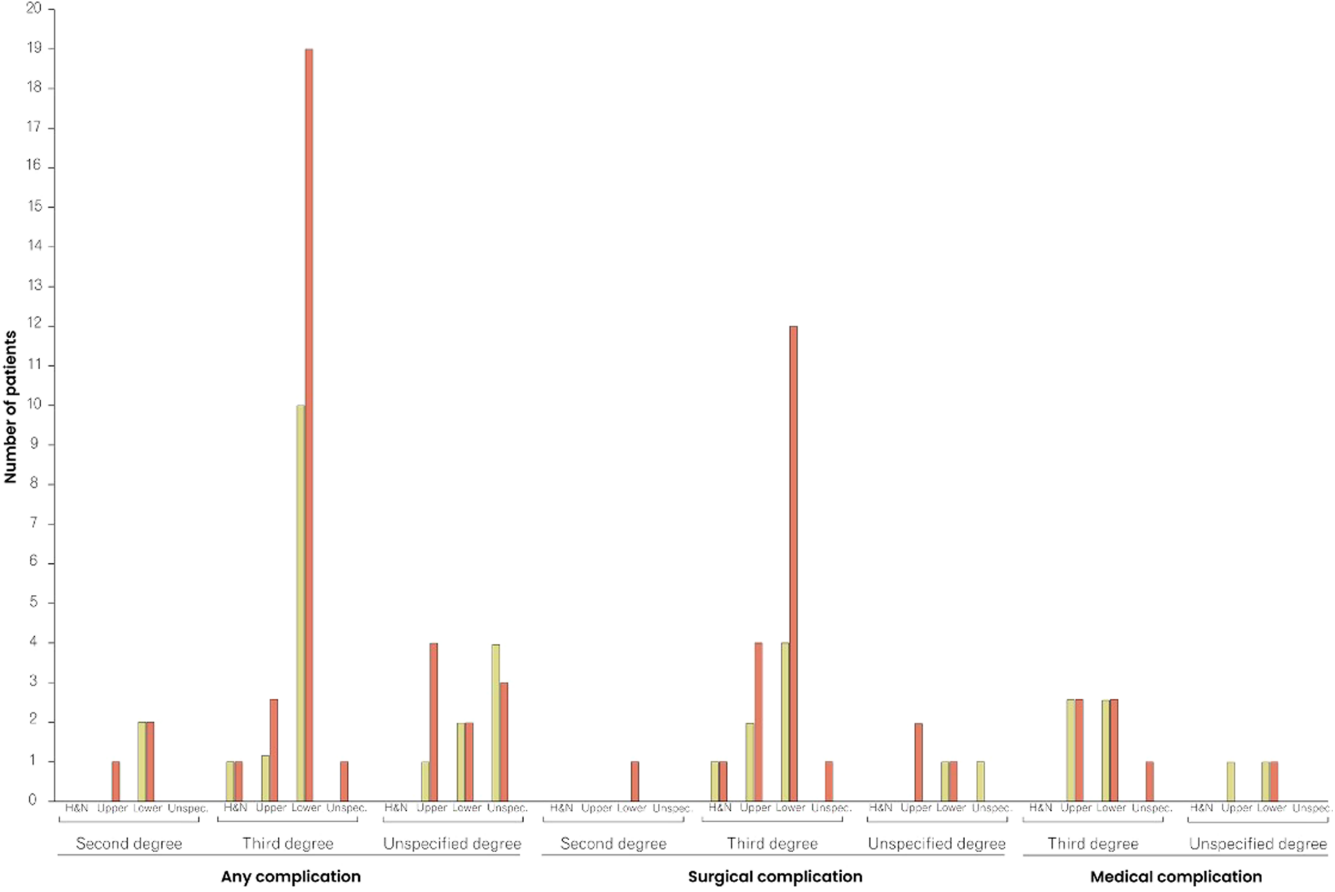
Comparison of complication rates between patients <60 and ≥60 years of age, stratified by type of complication, burn degree, and affected area. The red columns represent the frequency of complications in patients ≥60 years of age, and the yellow columns in patients <60 years of age. H&N, Head and Neck.

One (0.2%) death was reported within the 30-day follow-up period and 22 (4.8%) patients returned to the operating room. Readmission and unplanned readmissions were indicated in 26 (5.7%) and 12 (2.6%) cases, respectively. Any adverse events, including mortality, reoperation, (unplanned) readmission, surgical and medical complications, were reported in 74 (16%) cases. Surgical complications occurred in 31 (6.7%) cases, with superficial incisional infections (*n* = 10; 2.2%) and blood transfusions (*n* = 13; 2.8%) being the most common surgical complications. While seven (6.5%) of the 108 patients aged ≥60 years received blood transfusions, 352 patients <60 years of age received only six (1.7%) blood transfusions, marking a significant difference (*p* = 0.02) in the age-stratified incidence of transfusions. The rate of medical complications differed significantly (*p* = 0.04) between patients aged <60 years and those ≥60 years: in both groups, six cases of medical complications (1.7% and 5.6%, respectively) were reported. Table 3 provides detailed information on postoperative outcomes and complications. In both patients < and ≥60 years of age, any complications, surgical and medical complications were relatively and absolutely most frequent among patients with third-degree burns of the upper and lower body (Supplementary Table S2 and Figure 2).

### 3.5. Risk factors for complications

Advanced age (*p* < 0.0001), diabetes mellitus (*p* = 0.003), hypertension (*p* = 0.01), wound infection (*p* = 0.0003), higher ASA scores (*p* < 0.0001), and higher wound classes (*p* = 0.001) were identified as risk factors for the occurrence of any complication. Similarly, advanced age (*p* = 0.01), hypertension (*p* = 0.04), higher ASA scores (*p* < 0.0001), and higher wound classes (*p* = 0.0008) were predictors of surgical complications. For medical complications, again, advanced age (*p* = 0.0007), higher ASA scores (*p* < 0.0001), higher wound classes (*p* = 0.01), and wound infection (*p* = 0.0007) were found to be risk factors. Significant correlations between the occurrence of any, surgical and medical complications and inpatient procedures were noted (*p* < 0.0001, *p* = 0.0006, and *p* = 0.0004, respectively). Vice versa, the multivariate analysis confirmed the association (OR = −0.11, 95% CI: −0.18 to −0.03; *p* = 0.004) between an outpatient setting and a lower occurrence of any complications. Multivariate analysis revealed dialysis (OR = 0.44, 95% CI: 9.15–0.74; *p* = 0.003) and sepsis (OR=0.22, 95% CI: 0.06–0.37; *p* = 0.006) as independent risk factors for the occurrence of any complications, with dialysis (OR=0.29, 95% CI: 0.08–0.50; *p* = 0.006) and sepsis (OR=0.09, 95% CI: 0.02–0.15; *p* = 0.02) being significantly associated with surgical and medical complications, respectively. Tables 4, 5 provide a detailed breakdown of the risk factors for adverse events and the multivariate assessment of any, surgical, and medical complications. Analyzing the preoperative laboratory values, we identified elevations of BUN (*p* > 0.0001), creatinine (*p* > 0.0001), WBC (*p* = 0.02), and PTT (*p* = 0.004), as well as decreases of albumin (*p* = 0.0009) and hematocrit (*p* > 0.0001) as risk factors for the occurrence of any complications. Further details regarding the predictive value of preoperative lab parameters are described in Table 6.

**Table 4:** Risk factors for complications. Reported as *n* (%), unless otherwise stated.

Characteristic	Any complication		*p-*value	Surgical complication		*p*-value	Medical complication		*p-*value
	Yes	No		Yes	No		Yes	No	
	(*n* = 74)	(*n* = 386)		(*n* = 31)	(*n* = 429)		(*n* = 12)	(*n* = 448)	
Demographics									
Sex									
Female	25 (34)	152 (39)	0.36	12 (39)	165 (38)	0.98	4 (33)	173 (39)	0.77
Male	49 (65)	234 (61)		19 (61)	264 (62)		8 (67)	275 (61)	
Age, mean ± SD	53 ± 18	45 ± 16	**<0.0001**	53 ± 16	45 ± 17	**0.01**	62 ± 18	45 ± 17	**0.0007**
BMI, mean ± SD	31 ± 7.3	29 ± 7.5	0.12	30 ± 8.6	29 ± 7.4	0.61	32 ± 8.8	29 ± 7.5	0.22
Race			**0.02**			0.29			0.31
American Indian/Alaskan native	1 (1.4)	7 (1.8)		1 (3.2)	7 (1.6)		0 (0.0)	8 (1.6)	
Asian	0 (0.0)	9 (2.3)		0 (0.0)	9 (2.1)		0 (0.0)	9 (1.8)	
Native Hawaiian/Pacific Islander	1 (1.4)	52 (14)		0 (0.0)	53 (12)		0 (0.0)	53 (11)	
Black/African American	13 (18)	63 (16)		6 (19)	70 (16)		0 (0.0)	76 (16)	
White	57 (77)	272 (71)		23 (74)	306 (71)		10 (83)	319 (65)	
Other or unknown	2 (2.7)	33 (8.5)		1 (3.2)	34 (7.9)		2 (17)	33 (6.8)	
Setting			**<0.0001**			**0.0006**			**0.0004**
Outpatient	15 (20)	211 (55)		6 (19)	220 (51)		0 (0.0)	226 (46)	
Inpatient	59 (78)	175 (45)		25 (81)	209 (49)		12 (100)	222 (46)	
Preop health/comorbidities									
Diabetes	24 (32)	64 (17)	**0.003**	9 (29)	79 (18)	0.16	4 (33)	84 (17)	0.26
Insulin treated diabetes	12 (16)	35 (9.1)	**0.09**	6 (19)	41 (9.6)	0.11	3 (25)	44 (9.0)	0.11
COPD	1 (1.4)	9 (2.3)	>0.99	1 (3.2)	9 (2.1)	0.51	0 (0.0)	10 (2.0)	>0.99
Obesity	32 (43)	138 (36)	0.24	10 (32)	160 (37)	0.70	5 (42)	165 (34)	0.77
Hypertension	33 (45)	113 (29)	**0.01**	15 (48)	131 (31)	**0.04**	5 (42)	141 (29)	0.53
Dyspnea	3 (4.1)	10 (2.6)	0.45	2 (6.5)	11 (2.6)	0.22	0 (0.0)	13 (2.7)	>0.99
Disseminated Cancer	0 (0.0)	1 (0.3)	>0.99	0 (0.0)	1 (0.2)	>0.99	0 (0.0)	1 (0.2)	>0.99
CHF	2 (2.7)	2 (0.5)	0.12	1 (3.2)	3 (0.7)	0.24	0 (0.0)	4 (0.8)	>0.99
Current smoker	26 (35)	126 (33)	0.69	9 (29)	143 (33)	0.70	3 (25)	149 (31)	0.76
Corticosteroid use	1 (1.4)	10 (2.6)	>0.99	0 (0.0)	11 (2.6)	>0.99	0 (0.0)	11 (2.3)	>0.99
Wound infection	47 (64)	155 (40)	**0.0003**	19 (61)	183 (43)	0.06	11 (92)	191 (39)	**0.0007**
ASA class			**<0.0001**			**<0.0001**			**<0.0001**
1 – No disturbance	2 (2.7)	45 (12)		1 (3.2)	46 (11)		0 (0.0)	47 (9.6)	
2 – Mild disturbance	32 (43)	225 (58)		11 (36)	246 (57)		3 (25)	254 (52)	
3 – Severe disturbance	29 (39)	101 (26)		13 (42)	117 (27)		6 (50)	124 (25)	
4 – Life-threatening	9 (12)	13 (3.4)		5 (16)	17 (4.0)		2 (17)	20 (4.1)	
5 – Moribund	1 (1.4)	0 (0.0)		1 (3.2)	0 (0.0)		1 (8.3)	0 (0.0)	
Wound class			**0.001**			**0.0008**			**0.01**
1 – Clean	9 (12)	85 (22)		3 (9.7)	91 (21)		0 (0.0)	94 (19)	
2 – Clean/Contaminated	11 (15)	101 (26)		3 (9.7)	109 (25)		3 (25)	109 (24)	
3 – Contaminated	21 (28)	108 (28)		7 (23)	122 (28)		1 (8.3)	128 (29)	
4 – Dirty/Infected	33 (45)	92 (24)		18 (58)	107 (25)		8 (67)	117 (26)	
Functional Status			0.07			0.65			0.12
Independent	65 (88)	364 (94)		27 (87)	402 (94)		10 (83)	419 (86)	
Partially/Totally Dependent	7 (9.5)	16 (4.1)		2 (6.5)	21 (5)		2 (17)	21 (4.3)	

Statistically significant *p*-values are highlighted in bold.

**Table 5:** Multivariate assessment of any, surgical or medical complication occurrence for all patients undergoing burn surgery.

Risk factors	OR	95% CI	*P* value
Any complications			
Currently undergoing dialysis	0.44	0.15–0.74	0.003
Sepsis	0.22	0.06–0.37	0.006
Outpatient	−0.11	−0.18 to −0.03	0.004
Surgical complications			
Currently undergoing dialysis	0.29	0.08–0.50	0.006
ASA Class 5	1.02	0.41–1.62	0.001
Wound class^a^	0.03	0.00–0.05	0.03
Medical complications			
Sepsis	0.09	0.02–0.15	0.02
ASA Class 5	0.98	0.60–1.37	<0.0001

^a^ Direct correlation between ascending wound class (1–4) and complication occurrence.

**Table 6:** Preoperative laboratory values (reported as mean values with standard deviation) and their association with the occurrence of any complication.

Laboratory Value	Any complication		*P* value	Reference Range
	Yes	No		
	(*n* = 74)	(*n* = 386)		
Serum sodium (mmol/L)	138.5 (3.6)	138.2 (3.1)	0.82	135–145 mmol/L
BUN (mg/dl)	20.7 (15.8)	14.2 (6.6)	**>0.0001**	8–25 mg/dl
Creatinine (g/D)	1.7 (2.1)	1.0 (0.8)	**>0.0001**	F 0.6–1.8, M 0.8–2.4 g/D
Serum albumin (g/dl)	3.2 (0.7)	3.6 (0.7)	**0.0009**	3.1–4.3 g/dl
Total bilirubin (mg/dl)	0.7 (0.7)	0.6 (0.4)	0.12	0–1 mg/dl
SGOT (U/L)	43.1 (67.9)	32.8 (43.8)	0.25	F 9–25, M 10–40 U/L
Alkaline phosphatase (U/L)	97.5 (68.7)	90.6 (55.2)	0.51	F 30–100 U/L
WBC (×10^3^/mm^3^)	10.1 (4.6)	9.0 (3.2)	**0.02**	4.5–11 × 10^3^/mm^3^
Hematocrit (% of RBCs)	34.3 (6.5)	39.0 (5.4)	**>0.0001**	F 36.0–46.0%, M 37.0–49.0% of RBCs
Platelet count (×10^3^/µl)	268.6 (130.5)	273.6 (104.2)	0.75	130–400 × 10^3^/µl
PTT (sec)	36.3 (10.8)	31.1 (7.7)	**0.004**	25–35 s
INR of PT values	1.2 (0.2)	1.1 (0.5)	0.46	<1.1
PT (sec)	16.0 (4.7)	13.0 (2.1)	0.05	10–13 s

Statistically significant *p*-values are highlighted in bold.

BUN, blood urea nitrogen; SGOT, serum glutamic-oxaloacetic transaminase; WBC, white blood cells; PTT, partial thromboplastin time; INR, international normalized ratio; PT, prothrombin time; Min, minutes; SD, standard deviation; LOS, length of hospital stay; D, days; RBC, red blood cell.

## 4. Discussion

Most studies examining burn patients focus exclusively on outcomes in critically ill patients, with extensive and major burn injuries. Thus, there is a paucity of studies that investigate minor burn surgery holistically—across varying institutions, settings, and age groups. To address this knowledge gap, we queried the ACS-NSQIP multi-institutional database, analyzing early outcomes, medical and surgical complications, and predictive risk factors in 460 burn surgery cases. We included burn injuries of different degrees and stratified based on age, that were treated in both inpatient and outpatient settings. Albeit the %TBSA was not specified in the ACS-NSQIP database, a large body of evidence indicates that the vast majority of our patient cohort suffered minor burns with <10% TBSA: Namely, (i) the average operation time of less than one hour, (ii) the comparatively low mortality and morbidity, (iii) the relatively short LOS, and (iv) the nearly equal number of in- and outpatient care support the hypothesis of predominantly minor burn injuries (8, 15, 22–26).

With this assumption in mind, it was intriguing to explore the role of patient age in such non-extensive burns. Furthermore, as mentioned at the beginning, minor burns may lead to major harm. It remains, therefore, essential to identify—also among patients with minor burn injuries—specific perioperative risk factors.

### 4.1. Age as a risk factor for complications in burn surgery patients with minor burn injuries

In this study, 23% of all included patients were ≥60 years old. This proportion generally corresponds with burn injury demographics in the United States, where geriatric burns account for 20% of all burn patients (27). While elderly patients accounted for only about one-fourth of the patient cohort in our study, more than half of all observed complications occurred in patients aged ≥60 years (Table 3). Accordingly, our analysis revealed that patients ≥60 years of age were at significantly higher risk of any complications, with particular proneness to bleeding/blood transfusions and medical adverse events. Further, elderly patients were discharged more frequently to non-home skilled facilities.

A 2020 nationwide study from the Netherlands study found a considerably higher risk of morbidity, mortality, and decreased functional status, requiring facility discharge in patients over 65 years old after surgical burn management (15). Cobert et al. analyzed non-fatal burn hospitalizations in older adults concluding that higher age was predictive of discharge to non-independent living such as nursing homes or rehabilitation centers (28). While our findings generally align with previous reports, our work also revealed elevated complication incidences and non-home discharges in elderly burn surgery patients with minor, less-drastic injuries.

In addition, in our analysis, burn patients aged ≥60 years showed a significantly prolonged LOS, with a surplus of five LOS days on average. An Iranian study including 899 hospitalized burn patients reported a mean LOS of 3.2 days, while Lundy et al. identified advanced age as a risk factor for prolonged LOS (29, 30). While we found a slightly decreased LOS in our study cohort with predominantly minor burn injuries, we could confirm the predisposing role of age for prolonged LOS, with patients ≥60 years reporting a median LOS of 6 days. This finding carries high clinical relevance as prolonged LOS is correlated with increased costs and risk of nosocomial infections (31, 32).

Ultimately, the American Burn Association advises that elderly and other high-risk patients be treated for burns in burn centers, when possible, to receive specialized care and reduce complications. Although 35 of the 123 American burn centers (Supplementary Table S1) currently report patient data to the NSQIP (which includes 707 institutions), only a handful of analyses published by American burn centers investigate post-burn complications in the elderly (33, 34). Analyzing a cohort of 644 patients, Iles et al. found that elderly patients were most likely to have higher frailty, higher LOS, morbidity, and non-home discharge. They also reported an increased mortality rate in elderly patients, the majority of which suffered from minor burns (35). Similarly, in their prospective study of burn patients aged ≥65 years, Maxwell et al. noted that frail and elderly patients suffered from significantly more complications, such as sepsis, and higher mortality (36). Our findings of higher morbidity and complication rates, LOS, and non-home discharge agree with these reports. The increased incidence of such adverse events may be due to a higher prevalence of comorbidities such as diabetes and hypertension and an overall worse preoperative health status among patients aged 60 years and older (Table 1). For example, the calculated increased risk of bleeding/blood transfusion in the elderly patient cohort is likely a consequence of the lower hematological and coagulation values. Yet, underlying causalities need to be investigated more accurately in further large-scale studies.

Strikingly, while our univariate analysis highlighted age as a significant risk factor for any complication, a confounder-eliminating multivariate analysis demonstrated that age was no longer a risk factor (Tables 4, 5). Instead, the multivariate analysis revealed significant correlations between the occurrence of any complications and current dialysis treatment (OR = 0.44, 95% CI: 9.15–0.74; *p* = 0.003) and history of sepsis (OR = 0.22, 95% CI: 0.06–0.37; *p* = 0.006). Taken together, our findings point to a predisposing role of age in minor burn injuries and identify patients aged ≥60 years as particularly susceptible to adverse events. However, surgeons should be wary of focusing on the patient's age as an isolated clinical variable and rather also account for the surrounding comorbidities and concomitant treatment modalities. These findings also call for a re-thinking of the prevailing understanding that mere biological age is intrinsically associated with increased risk in burn surgery. The deliberation of patient eligibility, preoperative planning, and perioperative monitoring should be decoupled from the mono-perspective age consideration and replaced with a holistic view of the patient's characteristics.

### 4.2. Minor lower body burns as risk factor for any complication

When analyzing the affected burn area, and its association with postoperative complications, the lower body appeared to be a risk factor for both patients aged <60 years and ≥60 years (Figure 2 and Supplementary Table S2). Indeed, we demonstrated that minor lower body burns accounted for the largest proportion of all cases with any complication. Yeong et al. found an increased rate of tissue-expansion failure in burn patients with an affection of the lower limbs (37). Remarkably, Momeni et al. analyzed 995 burn cases and calculated that with every 1% increase in lower extremity burn surface area, the mortality increased by 9%. This statistical link was even more evident in elderly patients (38). Thus, while the lower body has previously been identified as a risk site, our study confirmed this observation for non-extensive burn lesions (39).

### 4.3. Laboratory parameters as predictive risk factors in burn surgery

We found markedly decreased serum albumin and hematocrit levels as well as elevated BUN, creatinine, and WBC levels in burn patients experiencing any postoperative complication. Further, the PTT was prolonged in patients suffering any complication. Previously, lower preoperative levels of hematocrit have been shown to correlate with an increased risk of intraoperative bleeding and the need for blood transfusion (40, 41). Hypoalbuminemia was found to worsen postoperative wound healing and negatively affect surgical site infections, while elevated BUN, creatinine, and WBC levels, and prolonged PTT have been linked with an increased risk of postoperative complications in general surgery and intensive care (42–50). Of note, albumin and hematocrit levels are generally lower in geriatric patients, whereas the same patient population has shown elevated BUN, and creatinine levels as well as a higher PTT (51–58). Accordingly, these risk-associated laboratory values may be considered proxy indicators of senescence, thereby underscoring the clinical relevance of advanced age in surgical burn care. With our results in mind, we propose particular attention to be paid to (elderly) patients with out-of-range preoperative levels of the above biomarkers in anticipation of postoperative adverse events. In addition, we suggest incorporating these laboratory values into frailty scores for burn surgery to further improve their sensitivity and specificity.

Ultimately, surgical interventions utilized for the treatment of burns in older patients remain a field of controversy and ongoing debate. Clinicians should take into consideration a variety of factors when deciding what surgical intervention to choose, especially when treating geriatric burns. Generally speaking, first-degree burns do not require surgical management, while deep second and third-degree burns often necessitate excision and grafting. However, the dermis in elderly patients is comparably thinner, which delays wound healing and may complicate harvesting and re-harvesting skin donor sites (59). Additionally, a well-vascularized wound bed is necessary for successful grafting, and older patients, especially those suffering from peripheral vascular disease, diabetes mellitus, or limb ischemia, are at higher risk for graft failure (60). Many surgeons contend that aggressive, early excision of deeply burned tissues and early grafting using an autologous split-thickness skin graft or synthetic skin replacement is essential in older patients to decrease infections, hospital stay, and accelerate recovery (15, 61, 62). While other clinicians advocate for a more conservative and delayed treatment approach to avoid surgical complications, previous reports have shown that early surgery in the elderly is better tolerated and leads to fewer complications (63, 64). Clinicians should consider the physiological changes that accompany older age, as well as the predictive risk factors and preoperative lab values mentioned in this study when evaluating a patient's eligibility for surgery and optimizing surgical outcomes and postoperative care. Furthermore, burn surgeons should also be aware that minor burn lesions may cause major damage and, therefore, require adequate care—particularly in the advanced patient age.

### 4.4. Limitations

Due to the multi-institutional nature of the ACS-NSQIP database, the analyzed patient pool can be considered relatively diverse and large. However, the inherent limitations should be considered. General limitations include the retrospective structure of the NSQIP database, which is innately subject to confounding factors and bias. Herein, we report only the presence of statistical correlations, while underlying causal-effect relationships need to be investigated in future studies. The accuracy and reliability of the records depend on the party responsible for data capture. Therefore, varying personal expertise and subjective rating may explain differing assessments, for example regarding the wound classification. In addition, the scope of the database has also been suggested as a potential source of bias, as there may be variability in quality both between and within the participating institution. Nonetheless, when assessing the data quality and interrater-reliability of the ACS-NSQIP database, Shiloach et al. found little variance in the heterogeneity of the catalog (65). Further, the standardized data collection results in a lack of potentially relevant information. The ACS-NSQIP database misses details on the patients' preoperative health (such as history of history of ischemic heart disease, coronary artery disease, or peripheral vascular disease) as well as on short-term (<30 days) procedure-specific complications including hematoma and edema. The postoperative follow-up is limited to 30 days, leaving long-term (>30 days) complications uncovered. The long-term success of burn surgery also depends on functional factors, such as pain and sensation, and aesthetic aspects, such as the presence and appearance of scars. These parameters are not included in the database. Similarly, the discharge destination may be biased by pre-admission domicile (at home or in a care facility), which is not reported and, therefore, not taken into account. The ACS-NSQIP database does not record information on the severity and chronicity of the comorbidities. Moreover, the burned body area (%TBSA), a crucial parameter in burn care, is not reported. Therefore, no relationship between the extent of burn and postoperative complications can be established in this study. Yet, we could estimate the extent of burn wounds in our patient cohort by considering the operative time, mortality and morbidity, LOS, and in- and outpatient distribution. In addition, in some cases, due to the limited information provided in the ACS-NSQIP database, the affected body region could not be clearly defined and was, therefore, left unspecified. One may speculate that patients who had suffered burns to multiple body regions may be in this category of “unspecified area”.

### 4.5. Conclusion

In this study, we utilized the NSQIP-ACS database to characterize patients receiving initial surgical care for predominately minor burn injuries. We also identified predictive risk factors for perioperative and postoperative complications, such as age and involvement of lower body parts. Although our multivariate analysis delineated age as a non-risk factor for complications, age can be a predictive risk factor when considering other coinciding factors such as dialysis treatment and sepsis. We demonstrated the relevance of preoperative laboratory values such as serum albumin and hematocrit in predicting postoperative adverse events. By including these novel variables and insights in the perioperative algorithm, surgeons may improve overall patient care and surgical outcomes in burn surgery.

## Data Availability

The datasets presented in this article are not readily available because formal restrictions apply to the availability of these data. Requests to access the datasets should be directed to American College of Surgeons—National Surgical Quality Improvement Program, https://accreditation.facs.org/programs/nsqip.
