# Supplementary material for: Association of age with perioperative morbidity among patients undergoing surgical management of minor burns
Source: Front Surg. 2023 Feb 27;10:1131293. doi: 10.3389/fsurg.2023.1131293 (PMC10008887; doi:10.3389/fsurg.2023.1131293)
Supplement: Supplementary file 1 [file Table1.pdf]

Supplementary Material

Association of Age with Perioperative Morbidity among Patients undergoing Surgical Management of Minor Burns

Samuel Knoedler*, Dany Y. Matar, Leonard Knoedler, Doha Obed, Valentin Haug, Sabina M. Gorski, Bong-Sung Kim, Martin Kauke-Navarro, Ulrich Kneser, Adriana C. Panayi, Dennis P. Orgill, Gabriel Hundeshagen*

*** Correspondence:**
samuel.knoedler@stud.uni-regensburg.de
gabriel.hundeshagen@bgu-ludwigshafen.de

Supplementary Table 1. Overlap between the participating ACS-NSQIP institutions and burn centers certified by the American Burn Association.

Banner University Medical Center - Phoenix
Barnes-Jewish Hospital at Washington University Medical Center
Baton Rouge General Medical Center - BlueBonnet Campus
Brigham & Women's Hospital
Dallas County Hospital District d/b/a Parkland Health
Erie County Medical Center Corporation
Grady Memorial Hospital
Indiana University Health - University Hospital
Jackson Memorial Hospital
Jacobi Medical Center
Johns Hopkins Hospital
Maine Medical Center
Memorial Hermann Hospital - TMC
Mercy Hospital South
North Carolina Baptist Hospital d/b/a Wake Forest Baptist Medical Center
Orlando Regional Medical Center
Our Lady of Lourdes Memorial Hospital
Rhode Island Hospital
Saint Joseph Hospital Health Center
Santa Clara Valley Medical Center
Spectrum Health Hospitals
Straub Clinic and Hospital
SUNY at Stony Brook University Hospital
The Ohio State University Wexner Medical Center
The University of Vermont Medical Center (aka Fletcher Allen Health Care - Hospital)
Thomas Jefferson University Hospital
University of Alabama at Birmingham Hospital
University of California San Diego Medical Center
University of Cincinnati Medical Center LLC
University of Iowa Hospitals and Clinics
University of Louisville Hospital
University of Rochester Medical Center
University of Utah Hospitals and Clinics
Vanderbilt University Medical Center
Wellstar Cobb Hospital
