# Supplementary material for: Association of age with perioperative morbidity among patients undergoing surgical management of minor burns
Source: Front Surg. 2023 Feb 27;10:1131293. doi: 10.3389/fsurg.2023.1131293 (PMC10008887; doi:10.3389/fsurg.2023.1131293)
Supplement: Supplementary file 2 [file Table2.pdf]

Supplementary Material

Association of Age with Perioperative Morbidity among Patients undergoing Surgical Management of Minor Burns

Samuel Knoedler*, Dany Y. Matar, Leonard Knoedler, Doha Obed, Valentin Haug, Sabina M. Gorski, Bong-Sung Kim, Martin Kauke-Navarro, Ulrich Kneser, Adriana C. Panayi, Dennis P. Orgill, Gabriel Hundeshagen*

*** Correspondence:**
samuel.knoedler@stud.uni-regensburg.de
gabriel.hundeshagen@bgu-ludwigshafen.de

Supplementary Table 2. Comparison of complication rates between patients younger than 60 and patients older than 60 years of age.

	Any complication		Surgical complication		Medical complication	
**Characteristic**	**≥60 Years (n = 25)**	**<60 Years (n = 49)**	**≥60 Years (n = 9)**	**<60 Years (n = 22)**	**≥60 Years (n = 6)**	**<60 Years (n = 6)**
**First Degree Burn**	0 (0.0)	0 (0.0)	0 (0.0)	0 (0.0)	0 (0.0)	0 (0.0)
**Second Degree Burn**						
Head and Neck	0 (0.0)	0 (0.0)	0 (0.0)	0 (0.0)	0 (0.0)	0 (0.0)
Upper Body	0 (0.0)	1 (2.0)	0 (0.0)	0 (0.0)	0 (0.0)	0 (0.0)
Lower Body	2 (8.0)	2 (4.1)	0 (0.0)	1 (4.5)	0 (0.0)	0 (0.0)
Unspecified Area	0 (0.0)	0 (0.0)	0 (0.0)	0 (0.0)	0 (0.0)	0 (0.0)
**Third Degree Burn**						
Head and Neck	1 (4.0)	1 (2.0)	1 (11)	1 (4.5)	0 (0.0)	0 (0.0)
Upper Body	5 (20)	16 (33)	2 (22)	4 (18)	2 (33)	2 (33)
Lower Body	10 (40)	19 (39)	4 (44)	12 (55)	2 (33)	2 (33)
Unspecified Area	0 (0.0)	1 (2.0)	0 (0.0)	1 (4.5)	0 (0.0)	1 (17)
**Unspecified Degree Burn**						
Head and Neck	0 (0.0)	0 (0.0)	0 (0.0)	0 (0.0)	0 (0.0)	0 (0.0)
Upper Body	1 (4.0)	4 (8.1)	0 (0.0)	2 (9.1)	1 (17)	0 (0.0)
Lower Body	2 (8.0)	2 (4.1)	1 (11)	1 (4.5)	1 (17)	1 (17)
Unspecified Area	4 (16)	3 (6.1)	1 (11)	0 (0.0)	0 (0.0)	0 (0.0)
